# How Well iPhones Measure Steps in Free-Living Conditions: Cross-Sectional Validation Study

**DOI:** 10.2196/10418

**Published:** 2019-01-09

**Authors:** Shiho Amagasa, Masamitsu Kamada, Hiroyuki Sasai, Noritoshi Fukushima, Hiroyuki Kikuchi, I-Min Lee, Shigeru Inoue

**Affiliations:** 1 Department of Preventive Medicine and Public Health Tokyo Medical University Shinjuku-ku Japan; 2 Department of Social and Behavioral Sciences Harvard T.H. Chan School of Public Health Boston, MA United States; 3 Department of Health Sociology and Health Education School of Public Health, Graduate School of Medicine The University of Tokyo Bunkyo-ku Japan; 4 Department of Life Sciences Graduate School of Arts and Sciences The University of Tokyo Meguro-ku Japan; 5 Division of Preventive Medicine Brigham and Women’s Hospital Harvard Medical School Boston, MA United States; 6 Department of Epidemiology Harvard T.H. Chan School of Public Health Boston, MA United States

**Keywords:** mobile phone, step count, physical activity, pedometer, epidemiology, population, validation, free-living conditions

## Abstract

**Background:**

Smartphones have great potential for monitoring physical activity. Although a previous laboratory-based study reported that smartphone apps were accurate for tracking step counts, little evidence on their accuracy in free-living conditions currently exists.

**Objective:**

We aimed to investigate the accuracy of step counts measured using iPhone in the real world.

**Methods:**

We recruited a convenience sample of 54 adults (mean age 31 [SD 10] years) who owned an iPhone and analyzed data collected in 2016 and 2017. Step count was simultaneously measured using a validated pedometer (Kenz Lifecorder) and the iPhone. Participants were asked to carry and use their own iPhones as they typically would while wearing a pedometer on the waist for 7 consecutive days during waking hours. To assess the agreement between the two measurements, we calculated Spearman correlation coefficients and prepared a Bland-Altman plot.

**Results:**

The mean step count measured using the iPhone was 9253 (3787) steps per day, significantly lower by 12% (1277/10,530) than that measured using the pedometer, 10,530 (3490) steps per day (*P*<.001). The Spearman correlation coefficient between devices was 0.78 (*P*<.001). The largest underestimation of steps by the iPhone was observed among those who reported to have seldom carried their iPhones (seldom carry: mean −3036, SD 2990, steps/day; sometimes carry: mean −1424, SD 2619, steps/day; and almost always carry: mean −929, SD 1443, steps/day; *P* for linear trend=.08).

**Conclusions:**

Smartphones may be of practical use to individuals, clinicians, and researchers for monitoring physical activity. However, their data on step counts should be interpreted cautiously because of the possibility of underestimation due to noncarrying time.

## Introduction

Monitoring daily physical activity using smartphones may have a great potential for public health applications [1]. Althoff et al [1] described how step-determined physical activity is distributed using a large-scale database consisting of 68 million days from 717,527 people in 111 countries, automatically measured using iPhones. However, little evidence exists on their measurement accuracy [2-5]. It is unclear how accurately step counts can be tracked via built-in algorithms of smartphones in free-living conditions because the smartphones may not be “tethered” to an individual at all times. For example, Hekler et al [3] examined the validity of physical activity measurement by a custom app of Android phones against an accelerometer in free-living conditions and showed that smartphones appear to be acceptable for estimating physical activity time. However, participants were instructed to carry their smartphones and wear the accelerometers at the same time during waking hours. In another study, Duncan et al [5] assessed various iPhone models in free-living conditions, but they did not fully account for the frequency and location of iPhone carrying. In the real world, individuals vary considerably regarding how much they carry their smartphones with them. Therefore, we aimed to assess the accuracy of step counts measured using smartphones in free-living conditions, under typical conditions where the smartphones may not always be carried by the individuals, using the default installment of a step counter app on the iPhone, against a pedometer.

## Methods

### Study Sample

We recruited a convenience sample of 54 healthy adults (mean age 31, SD 10, years; 48%, 26/54, men) who owned an iPhone 5S, 6, 6S, 6plus, SE, or 7 (Apple Inc, California, United States) through direct outreach and flyers at a university in 2016 and 2017. Each participant received a 3000 Japanese Yen (US $25) gift card for participating in the study. Ethical approval was granted by Tokyo Medical University Ethics Committee.

### Measures

Daily step count was measured using both a validated pedometer, Kenz Lifecorder Ex (Suzuken Co, Ltd, Nagoya, Japan) [6,7], and an iPhone. Schneider et al, in their validation study using 13 pedometer models, have reported that Kenz Lifecorder Ex is suitable for most research purposes (compared to the criterion pedometer, Yamax SW-200), with an observed mean difference in the step count of −703 (SD 1537) steps per day [7]. We used the Health app preinstalled on the iPhone to measure steps using iPhone. Participants were asked to carry their own iPhones as usual and wear a pedometer on their waist for 7 consecutive days during waking hours. A self-reported questionnaire evaluated sociodemographic and health-related factors, as well as how (in their pockets or bags) and how often (almost always, sometimes, seldom) participants carried their iPhones. A record was deemed valid if the pedometer was worn for ≥10 hours a day [8,9] for at least 3 days [10].

### Statistical Analysis

The mean and SD of the step count for each device was obtained. We calculated Spearman correlation coefficients, intraclass correlation coefficient (ICC), and weighted kappa using a classification matrix. The difference in the step count between device measurements was calculated by subtracting the step count of the pedometer from that of the iPhone. A paired *t* test was performed to determine whether the differences between step counts were statistically significant. We performed a 2-sample *t* test and linear regression analysis to detect differences according to iPhone carrying locations and linear trend for frequency, respectively. An ordinal scale was used when the trend tests were run. A Bland-Altman plot was created to assess the agreement between the two measurements [11]. In sensitivity analysis, we included data only from participants with ≥13 hours of pedometer wear time [12,13]. Analyses were conducted in 2017 using IBM SPSS Statistics version 21 (IBM Corp).

## Results

**Figure 1:**
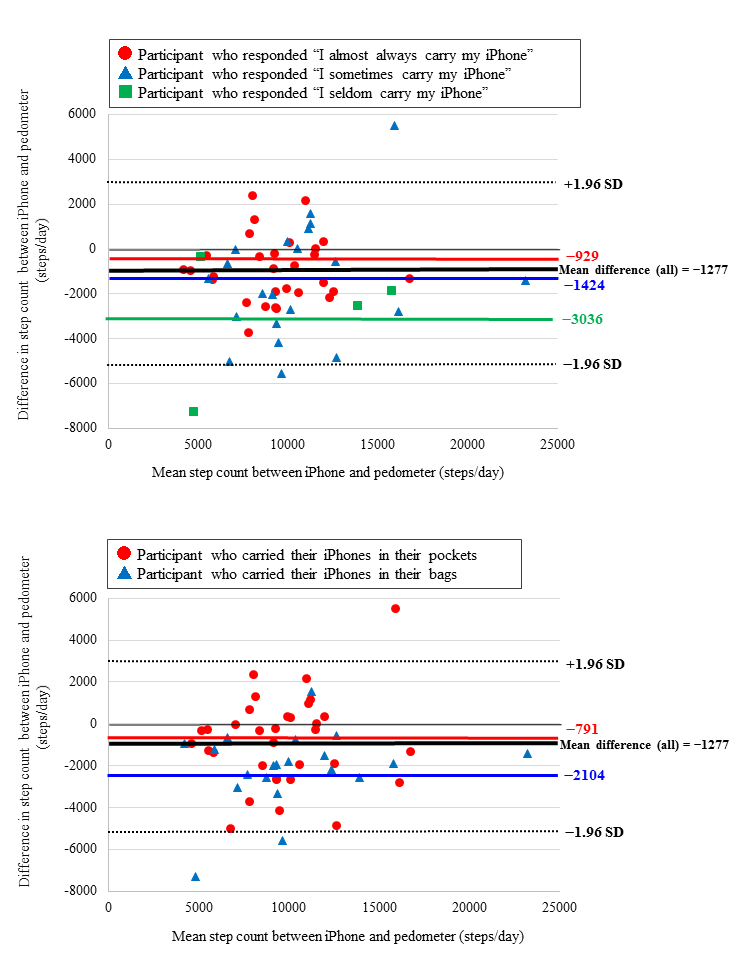
Comparison of daily steps measured using iPhone and pedometer in free-living conditions (N=54).

The mean step count measured using the iPhones was 9253 (SD 3787) steps per day; this was significantly lower than that measured using the pedometer, 10,530 (SD 3490) steps per day (mean relative difference 12% [SD 21%]; *P*<.001). Spearman correlation coefficient between the devices was 0.78 (*P*<.001), and ICC was 0.88 (95% CI 0.79-0.93; *P*<.001). When categorized into quartiles based on step count, the pedometer and iPhone classified participants into the same quartile 54% (29/54) of the time, resulting in a weighted kappa coefficient of 0.69. The Bland-Altman plot revealed a mean difference in step count of −1277 (SD 2122) steps per day, with no significant proportional bias (Figure 1).

In the first graph in Figure 1, the thick black line shows mean difference among overall sample; dotted black lines show mean (SD 1.96); red line shows mean difference among those who almost always carry their iPhone; blue line shows mean difference among those who sometimes carry their iPhone; and green line shows mean difference among those who seldom carry their iPhone. A negative difference value means the step count measured using the iPhone was lower than that measured using the pedometer (ie, underestimated). There was no significant proportional bias between the two methods (*r*=0.06). In the second graph, the thick black line shows mean difference among overall sample; dotted black lines show mean (SD 1.96); red line shows mean difference among those who carry their iPhone in their pockets; and blue line shows mean difference among those who carry their iPhone in their bags. A negative difference value means the step count measure using the iPhone was lower than that measured using the pedometer (ie, underestimated).

We then assessed whether step counts from smartphones may be sensitive to how frequently participants carried their iPhones with them (Figure 1). The largest underestimation of steps using the iPhones against the pedometer was observed among those who reported to have seldom carried their iPhones, with borderline statistical significance (seldom carry: −3036, SD 2990, steps/day; sometimes carry: −1424, SD 2619, steps/day; and almost always carry: −929, SD 1443, steps/day; *P* for linear trend=.08). Sensitivity analyses restricting the analyses to participants with ≥13 hours of pedometer wear time also yielded similar findings that were statistically significant (seldom carry: −3036, SD 2990, steps/day; sometimes carry: −1721, SD 2095, steps/day; and almost always carry: −1032, SD 1401, steps/day; *P* for linear trend=0.03). Additionally, step counts were more underestimated among participants who typically carried their iPhones in their bags (−2104, SD 1844, steps/day) than among those carrying the smartphones in their pockets (−791, SD 2149, steps/day; *P*=.02; Figure 1), although the tests for interaction of iPhone carrying location and frequency with the differences in step counts were not significant, possibly due to small sample sizes in the subgroups. There was no significant interaction of iPhone carrying location and frequency with differences in step count between the pedometer and iPhone.

When stratified by gender, difference in the step count between device measurements was larger among women than among men (−1847, SD 1880, steps/day vs −664, SD 2231, steps/day; *P*=.04; Table 1). Most (18/28, 64%) of the women carried their iPhones in their bags rather than in their pockets, whereas almost all (24/26, 92%) of the men carried them in their pockets (Table 2).

**Table 1:** Gender differences in daily steps measured using iPhone.

Characteristics		Men	Women	*P* value
Age in years, mean (SD)		30 (10)	32 (10)	.39^a^
**Steps measured using, mean (SD)**				
	Pedometer	9864 (3094)	11,149 (3770)	.18^a^
	iPhone	9200 (3332)	9302 (4227)	.92^a^
Differences between the two measurements (iPhone−pedometer steps), mean (SD)		−664 (2231)	−1847 (1880)	*.04* ^a,b^
**Usage of iPhone model, n (%)**				.87^c^
	5S	9 (35)	11 (39)	
	6	8 (31)	5 (18)	
	6S	6 (23)	8 (29)	
	SE	1 (4)	2 (7)	
	7	2 (8)	2 (7)	

^a^*P* value was calculated using *t* test.

^b^Italicized values indicate statistically significant differences.

^c^*P* value was calculated using Fisher Exact test.

**Table 2:** Gender differences in frequency and location of carrying an iPhone.

Characteristics		Men		Women		*P* value
		n (%)	Mean (SD)	n (%)	Mean (SD)	
**Frequency of carrying an iPhone**						.30^a^
	Almost always	17 (65)	−439 (1647)	12 (43)	−1623 (679)	*.03* ^b,c^
	Sometimes	8 (31)	−1181 (3336)	13 (46)	−1573 (2208)	.75^b^
	Seldom	1 (4)	N/A^d^	3 (11)	−3928 (2939)	N/A
**Location of carrying an iPhone**						*<.001* ^a^
	In the pocket	24 (92)	−573 (2289)	10 (36)	−1314 (1765)	.37^b^
	In a bag	2 (8)	−1757 (1151)	18 (64)	−2143 (1925)	.79^b^

^a^*P* value was calculated using Fisher Exact test.

^b^*P* value was calculated using *t* test.

^c^Italicized values indicate statistically significant differences.

^d^N/A: not applicable.

## Discussion

### Principal Findings

We found that step counts measured using a pedometer or iPhone correlated moderately well under free-living conditions. In contrast to a previous laboratory-based study where only a small difference in the mean step count between iPhone apps and direct observation was found [4], we found that iPhone underestimated average step count by 12% (1277/10,530) compared to a pedometer. These findings were similar to that of previous study where step counts measured using iPhone were underestimated by 1340 steps per day in free-living conditions [5]. Furthermore, the level of underestimation depended on how often participants typically carried the phone with them, as well as different carrying locations of the phone. To improve the accuracy of step counts measured using iPhones, carrying a phone as frequently as possible appears important.

With the growing popularity of smartphones [14], step counting apps make objective tracking of physical activity available to a tremendous number of people [1]. Smartphones may be of practical use to researchers for monitoring step-determined physical activity and for health promotion. Furthermore, clinicians can obtain a patient’s daily physical activity data immediately in clinical practice. However, investigators and clinicians also should be aware of the potential for underestimation of step counts using smartphones especially when the interest is in its between-individual variation, including country-level comparisons. For example, a previous study of step-determined physical activity for free-living individuals measured using an iPhone app identified inactive subpopulations such as women [1]. The finding that women took fewer steps than men regardless of age groups may partly be attributable to the phone carrying habits and location of phone carrying among women. In particular, women’s clothing, such as dresses, rarely have pockets large enough to fit a smartphone, and in our study, most women carried their iPhones in their bags rather than in their pockets.

The mean bias of step counts measured using iPhone slightly exceeded the ±10% “acceptable” difference range used in previous free-living studies [7,15]. In addition, limits of agreement ranged from −5436 to 2882 steps per day for all participants (−3757 to 1899 steps/day among those who almost always carried an iPhone). However, this difference is comparable to that observed for other pedometers that are considered acceptable for research purpose [7,15].

### Limitations

We investigated only healthy, young Japanese adults who were more active than the general population [16] and owned an iPhone; it is unclear whether our results are applicable to other individuals and other smartphone apps. In this study, there might have been an underestimation of differences in step counts between Kenz Lifecorder Ex and the iPhone. Although previous studies have found Kenz Lifecorder Ex to be acceptable compared to gold standard pedometers, the former may slightly underestimate step counts in free-living conditions [7]. Thus, the inherent technical measurement error of the pedometer used in this study is a limitation.

### Conclusions

We found that step count measured using a pedometer and iPhone correlated moderately well in free-living conditions. Smartphones can be of practical use to individuals, clinicians, and researchers for monitoring physical activity and for health promotion. However, their data on step counts should be interpreted cautiously because of the possibility of underestimation due to noncarrying time and carrying locations, as well as gender differences.

## Abbreviations

ICC: intraclass correlation coefficient
